# Supplementary material for: Novel Biological and Molecular Characterization in Radiopharmaceutical Preclinical Design
Source: J Clin Med. 2021 Oct 21;10(21):4850. doi: 10.3390/jcm10214850 (PMC8584913; doi:10.3390/jcm10214850)
Supplement: Supplementary file 1 [file jcm-10-04850-s001.zip › jcm-1410052-supplementary.pdf]

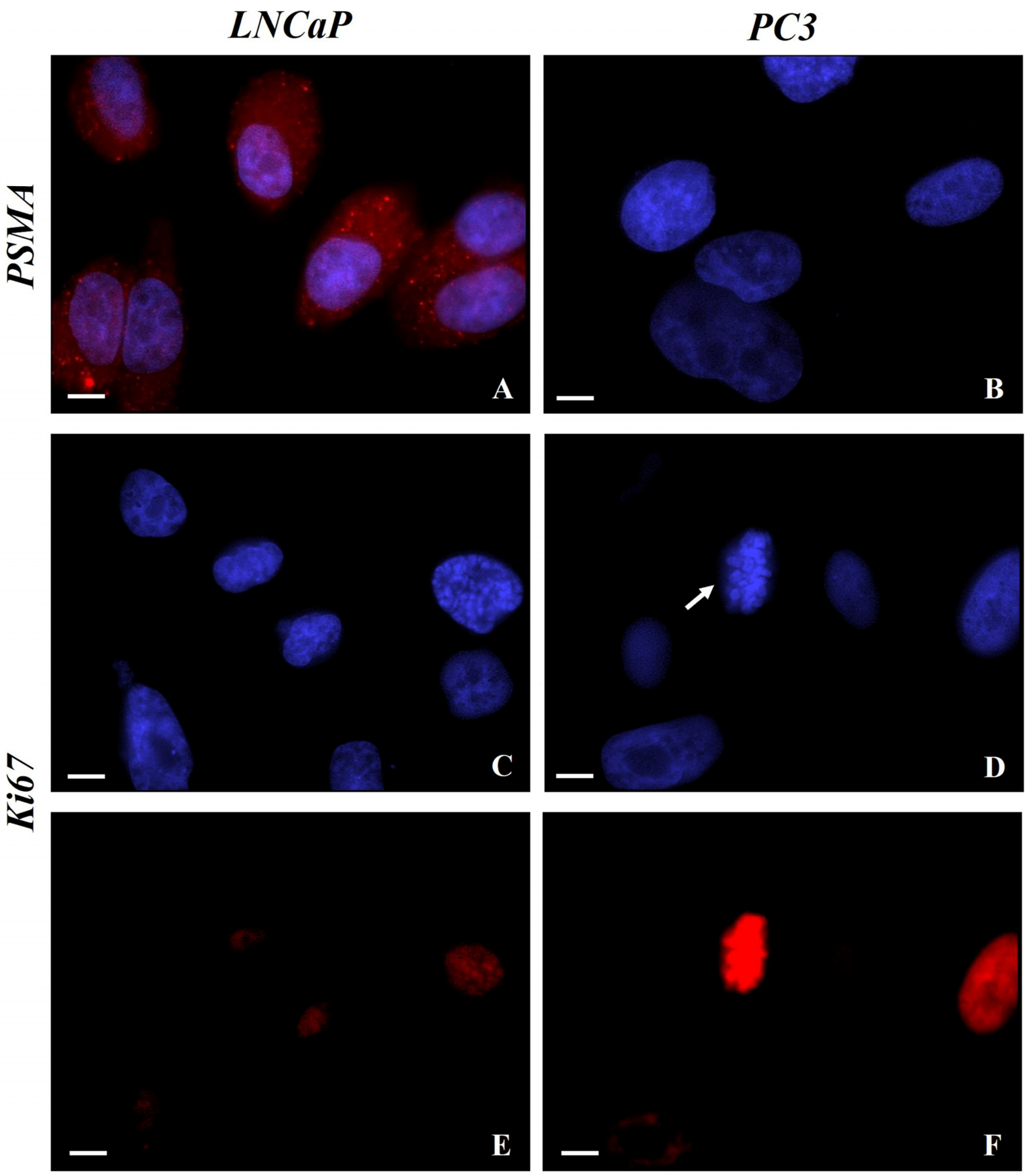

Figure S1. Cell Culture Immunoflurescence. A) Numerous PSMA positve LNCaP cells. B) Image shows no PSMA expression in PC3 cells. C) DAPI Staining of LNCaP cells. D) DAPI Staining of PC3 cells. Arrow mark a cell during the mitotic process. E) Texas Red staining displys Ki67 positive LNCaP cells. F) Texas Red staining displys Ki67 positive PC3 cells. Scale bar represents 50μm for all images.
